# Case report: Headache as the sole neurological symptom in autoimmune glial fibrillary acidic protein (GFAP) astrocytopathy

**DOI:** 10.3389/fneur.2024.1366263

**Published:** 2024-04-18

**Authors:** Eslam Shosha, Colleen Connolly, Adrian Budhram

**Affiliations:** ^1^Neurology Division, Department of Medicine, McMaster University, Hamilton Health Science Center, Hamilton, ON, Canada; ^2^Department of Clinical Neurological Sciences, Western University, London Health Science Center, London, ON, Canada; ^3^Department of Pathology and Laboratory Medicine, Western University, London Health Science Center, London, ON, Canada

**Keywords:** headache, meningoencephalitis, glial fibrillary acidic protein, astrocytopathy, GFAP

## Abstract

Autoimmune glial fibrillary acidic protein (GFAP) astrocytopathy is a recently emerging autoimmune disease of the central nervous system (CNS); GFAP astrocytopathy is characterized by optic neuritis and meningoencephalomyelitis. We report the case of a 55-year-old man, otherwise healthy, who presented with isolated headaches for three months, without other features of meningoencephalitis or myelitis. His neurological examination and fundoscopy were unremarkable. Gadolinium-enhanced brain MRI demonstrated increased T2 hyperintensity within the right sub-lenticular basal ganglia, with additional leptomeningeal enhancement along the bilateral perisylvian regions and mesial temporal lobes. Cerebrospinal fluid (CSF) analysis showed lymphocytic pleocytosis, elevated protein, matching oligoclonal bands, and a negative infectious and cytological workup. Cell-based assays for anti-aquaporin-4, anti-myelin oligodendrocyte glycoprotein, autoimmune encephalitis panel, and vasculitis workup were all negative, except for CSF positivity for GFAP α antibody. Oncological screening, including CT of the chest, abdomen, pelvis, and scrotal US, was unremarkable. Immunotherapy with high-dose intravenous steroids for five days and subsequent single four-weekly doses resulted in the resolution of both clinical and radiographic features, with a maintained status 24 months after onset. This case highlights isolated headache and basal ganglia, mesial temporal lobe involvement as a rare presentation of autoimmune GFAP astrocytopathy.

## Introduction

Autoimmune glial fibrillary acidic protein (GFAP) astrocytopathy is a recently described neural antibody-associated disease that typically presents with meningoencephalomyelitis or limited forms thereof (1–5). Diagnostic certainty is highest when positivity for antibodies against the GFAPα isoform (anti-GFAP) in CSF is demonstrated by tissue indirect immunofluorescence (TIIF) and cell-based assays in a patient with a compatible clinical phenotype (6, 7).

Here, we describe a case of autoimmune GFAP astrocytopathy with isolated headache and basal ganglia, mesial temporal lobe involvement that resolved entirely after administration of high-dose steroids.

### Case report

A healthy 55-year-old right-handed man presented to our clinic complaining of headaches for three months. The headache was throbbing and localized to the right hemi-cephalic region without any neck soreness. The pain was severe, with some variability during the day, and lasted for one week. The pain was refractory to paracetamol, ibuprofen, and intravenous analgesia during frequent visits to urgent care. Over three months, the intensity gradually decreased and was localized to the bi-parieto-occipital region. There were no constitutional symptoms, weight loss, weakness, sensory symptoms, Lhermitte’s, or evidence of bowel or bladder involvement. His neurological examination was unremarkable, including orientation to time and place, language assessment, cranial nerves, fundoscopy, motor, sensory, coordination, and gait. His physical examination was also normal.

A blood examination showed a white blood cell count in the normal range (8,300 /μL). His C-reactive protein, antinuclear antibody (ANA), extractable nuclear antigen (ENA), angiotensin-converting enzyme (ACE), Rheumatoid factor (RF), Anti-double-stranded deoxyribonucleic acid antibodies (ds-DNA), Anti-Neutrophilic Cytoplasmic Antibody (ANCA) and vasculitis markers, in addition to tumor markers including prostate-specific antigen (PSA), carcinoembryonic antigen (CEA), carbohydrate antigen (CA19-9), and human immunodeficiency virus (HIV) serology were normal. CSF analysis: The white blood cell count was 19 /μL (95% lymphocytes), the protein was 66 mg/Dl, the glucose level was 3 mmol /L; blood glucose was 5.2 mmol/L, and matched oligoclonal bands in both serum and CSF. Cytology, and infectious workup, including PCR for Herpes Simplex 1, 2, Enterovirus, West Nile virus, JC/ BK polyomaviruses, and Varicella Zoster virus were normal. Comprehensive neural antibody testing in serum and CSF was unremarkable, with the exception of positivity for anti-GFAP in CSF by TIIF and CBA (titer by TIIF 1:8, reference range < 1:2). Anti-GFAP was detected at the London Health Sciences Centre by TIIF performed as part of their comprehensive autoimmune encephalitis panel. Confirmatory testing for anti-GFAP by TIIF and CBA and titer by TIIF were performed at the Mayo Clinic.

**Figure 1:**
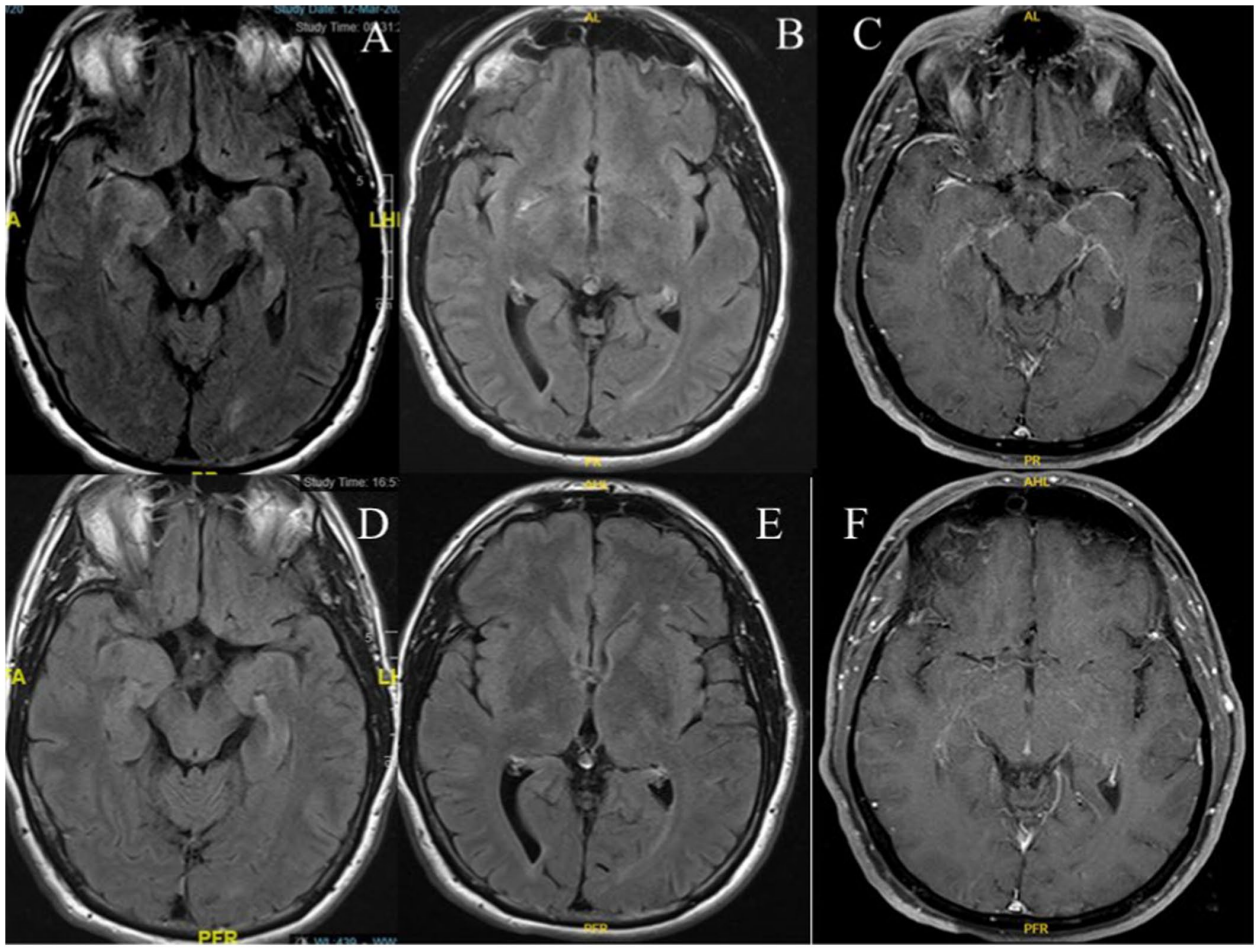
Brain MRI shows a T2 hyperintense lesion in the mesial temporal lobes **(A)**, right sub-lenticular region on axial FLAIR **(B)**, and gadolinium enhancement in the right>left leptomeningeal, perisylvian regions, and mesial temporal lobes on axial T1-weighted post-gadolinium administration **(C)**. Follow-up MRI shows resolution of findings on axial FLAIR **(D,E)**, and T1-weighted post-gadolinium administration **(F)**.

Brain MRI with gadolinium revealed T2 hyperintensity lesions in the right sub-lenticular region and gadolinium enhancement in the right>left leptomeningeal, perisylvian regions, and mesial temporal lobes (Figures 1A–C). A follow-up MRI was performed three months later and showed a resolution of the previous findings (Figures 1D–F). MRI of the spinal cord and CT of the chest, abdomen, and pelvis with contrast were unremarkable. Treatment was initiated while awaiting the results of the neural autoantibodies, and the patient was given intravenous methylprednisolone (1 g/day for five days) followed by 1 g weekly for four weeks. Significant clinical, radiological, and functional improvement was observed.

Two years after onset the patient remained asymptomatic with no relapses.

## Discussion

GFAP astrocytopathy is a rare autoimmune CNS disease that classically presents as meningoencephalomyelitis or limited forms thereof. Clinical phenotypes are varied but include brainstem/cerebellar dysfunction, altered mental status, movement disorders, cognitive decline, psychiatric symptoms, spinal cord and autonomic dysfunction, and visual tract and peripheral nervous system involvement (5, 7). While headache commonly occurs with other symptoms of meningoencephalomyelitis, headache as the sole clinical manifestation of autoimmune GFAP astrocytopathy, as we present here, is distinctly unusual (8–10).

The underlying pathogenesis of autoimmune GFAP astrocytopathy is diverse, including infectious, post-infectious, and autoimmune diseases of the CNS. Coexisting neural autoantibodies like antibodies to NMDA receptors, GABA-A receptors, and aquaporin-4 have been reported in some patients with anti-GFAP astrocytopathy (5, 6), but this was not found in our patient, nor was there evidence of a concomitant infectious process or sign of neoplasms. We found the cardinal feature of CSF positivity for GFAP α antibody (diagnostic for autoimmune GFAP astrocytopathy) (3, 9–12). Although not specific for autoimmune encephalitis (6), our patient’s CSF showed inflammatory parameters, an elevated protein level, and lymphocytic pleocytosis, which is a common finding in GFAP astrocytopathy (12) and supports our diagnosis. GFAP is the main intermediate filament protein in mature astrocytes and a cytoskeleton component. It is the main target of autoimmune GFAP astrocytopathy, which is thought to be caused by a cytotoxic response mediated by T cells (11). Since GFAP is an intracellular antigen, GFAP-IgGs are likely non-pathogenic intermediaries in the autoimmune response causing the disease (11).

Even though neuroimaging abnormalities have been found in only 50% of the reported patients (6), the findings in our patient are consistent with those consistently described in patients with autoimmune GFAP astrocytopathy; namely basal ganglia involvement in addition to linear perivascular radial gadolinium enhancement extending toward the cortex from the lateral ventricles on MRI (5, 7). This further supports the diagnosis.

In accordance with the standard protocol used in previous cases, our patient was treated with methylprednisolone boluses of 1 g daily for five days, and as in most reports there was a remarkable improvement in symptoms and results of investigations (5, 6, 11). Interestingly, this resolution occurred despite the three-month delay between symptom onset and diagnosis. Treatment in the acute stage usually involves intravenous administration of high-dose corticosteroids with substantial clinical improvement (11). However, intravenous immunoglobulin or plasma exchange is sometimes required to achieve disease remission (7). We did not feel it was necessary to use these modalities in our patient, although we continued to administer 1 g of methylprednisolone every week for six weeks to achieve complete symptom resolution. Long-term treatment with oral steroids or immunosuppressants such as mycophenolate mofetil, azathioprine, or rituximab is required in 20–50% of the cases to prevent relapses (13), but this was not deemed necessary in our patient.

## Conclusion

In summary, we highlight a unique, atypical clinical but not radiological delineation of autoimmune GFAP astrocytopathy. Much work is needed to clarify the nature and clinical features associated with this rare condition. The urgency of the diagnosis necessitates raising awareness among healthcare providers to consider this possibility in any patient presenting with an acute-onset persistent headache. Early identification of this treatable disease is essential to prevent neurological and functional deterioration due to its progression. However, it is a challenge to diagnose the disease in its early stages as these features overlap with many forms of autoimmune and infectious CNS disorders. Detection would be greatly improved if a more uniform diagnostic consensus could be formulated as to who should undergo testing for GFAP astrocytopathy. This is currently a challenge as the diagnosis is likely to be missed and the disease is underdiagnosed due to the sparse data available and the overlap of its clinical manifestations with other autoimmune and infectious disorders, especially in the early stages.

## Data availability statement

The raw data supporting the conclusions of this article will be made available by the authors, without undue reservation.

## Ethics statement

Ethical approval was not required for the study involving humans in accordance with the local legislation and institutional requirements. Written informed consent to participate in this study was not required from the participants or the participants’ legal guardians/next of kin in accordance with the national legislation and the institutional requirements. Written informed consent was obtained from the individual(s) for the publication of any potentially identifiable images or data included in this article.

## Author contributions

CC: Writing – review & editing. AB: Writing – review & editing. ES: Conceptualization, Writing original draft, Writing – review & editing, Supervision.
